# Factors associated with low vs increased perceived mastery of clinical work over ten years of practice: a prospective study of Norwegian doctors

**DOI:** 10.1186/s12909-018-1236-9

**Published:** 2018-05-29

**Authors:** Anna Belfrage, Kjersti Støen Grotmol, Reidar Tyssen, Torbjørn Moum, Lars Lien

**Affiliations:** 1 Norwegian National Advisory Unit on Concurrent Substance Abuse and Mental Health Disorders, Innlandet Hospital Trust, P.O. Box 104, 2381 Brumunddal, Norway; 2 Department of Behavioral Sciences in Medicine, Institute of Basic Medical Sciences, Faculty of Medicine, Medical Faculty, University of Oslo, P.O. Box 1111 Blindern, 0317 Oslo, Norway; 3 Regional Centre of Excellence in Palliative Care, Department of Oncology, Oslo University Hospital, Oslo, Norway; 4 Faculty of Public Health, Innlandet University College, Box 100, 2400 Elverum, Norway

**Keywords:** Perceived mastery of work, Physicians’ health, Leading position, Social support, Alcohol to cope

## Abstract

**Background:**

A higher sense of mastery of doctors’ clinical work could benefit not only their own mental health but also their work performance and patient care. However, we know little about factors associated with perceived mastery of clinical work among physicians. Our aim was therefore to study characteristics of those with stable low levels and of those with increased levels of mastery over a period of ten years of medical practice.

**Methods:**

*N* = 631 doctors were surveyed in their final year of medical school in 1993/94 (T1) and 10 (T2), 15 (T3) and 20 (T4) years later. Low and increased perceived mastery of clinical work were measured between T2, T3 and T4. Response rates for all items measuring low and increased mastery were 238/522 (46%) and 256/522 (49%) respectively. The following explanatory variables were included: demographics, medical school factors, personality and contextual work-related and non-work-related factors.

**Results:**

*N* = 73 (31%) of the doctors reported stable low mastery from T2 to T4. The following variables were significantly associated with low mastery in the adjusted analyses: *vulnerability* (OR: 1.30, *P* < .000, CI: 1.12 to 1.50), drinking *alcohol to cope* with stress during medical school (OR: 2.66, *P* = .04, CI: 1.03 to 6.85) and *social support* (OR: 0.78, *P* = .002, CI: 0.66 to 0.91). *N* = 39 (15%) reported increased mastery during the ten-year period from T2 to T4. Perceived *job demands* (OR: 0.66, *P* = .02, CI: 0.45 to 0.98) and taking up a *leading position* (OR: 3.04, *P* = .01, CI: 1.31 to 7.07) were associated with increased mastery after adjustment.

**Conclusions:**

Stable low sense of mastery over time is associated with having a vulnerable personality, a history of having used alcohol to cope with stress during medical school and lack of contemporary social support. Conversely, increased sense of mastery is associated with taking up a leading position and having the perception that job demands are decreasing over time. These findings indicate that perceived mastery of clinical work may not be a trait, but a state modifiable over time.

## Background

A sense of mastery in demanding or stressful situations might lead to personal development and resilience against mental distress [1, 2]. Physicians constitute a professional group exposed to many demanding and stressful situations [3], which require healthy coping strategies [4]. At least 10–30% of physicians suffer from symptoms of burnout [5, 6], and the number is increasing [3, 7]. A higher sense of mastery of their clinical work could benefit the physicians’ mental health, their work performance and the quality of their patient care [8, 9]. However, we know little about perceived mastery of clinical work among physicians, and to our knowledge there are no studies on long-term factors associated with perceived mastery in a representative sample of doctors.

Some previous cross-sectional studies on factors associated with mastery of work in other populations show that a high sense of job autonomy and control combined with an appropriate level of demands [10, 11] at work appears to increase sense of mastery, while job exhaustion, sense of need for recovery after work [12] and the personality trait of “neuroticism” [11] are associated with low mastery. Even colleague support [11], social support [1], and occupational prestige [13] have been shown to boost the sense of mastery of work.

There are studies showing that an active problem-solving behavior and a feeling of mastery in non-work-related situations may increase one’s sense of mastery at work [14, 15]. Drinking alcohol to cope with stress – which can be seen as a form of avoidant behavior – has predicted lower perceived mastery of clinical work among doctors in a previous study by our research group [16].

We believe that contextual factors, both work-related and non-work-related, that are somehow linked to ways of handling stress and high demands could have an impact on mastery. Being in a leading position at work could be seen as an example of having put oneself in a demanding situation, and working more hours per week could lead to more exposure and experience in clinical work, which ultimately might lead to sense of mastery. Since mastery of clinical work has an underlying component of social interaction (with patients) we believe that social interaction, such as colleague support or social support outside of work, can be important. The manageability component in Antonovsky’s concept of sense of coherence includes a belief in handling demanding situations on one’s own but also a belief that one can rely on close others (collegial or social support) if needed [17]. We do, however, need more research on the impact on perceived mastery among doctors of work-related and other contextual variables.

The aim of this study was therefore to investigate the influence of contextual work and non-work related factors on doctors’ perceived mastery of clinical work by asking the following questions: 1) How many doctors report *stable low* perceived mastery over a period of ten years of practice, and what characterizes them? 2) How many doctors report *increased* perceived mastery over a period of ten years of practice, and what characterizes them?

## Methods

### Participants and study design

We used data from the Young Doctor Cohort (*n* = 631) of the Longitudinal Study of Norwegian Medical Students and Doctors (NORDOC) [18]. The cohort consists of young doctors surveyed with postal questionnaires over 20 years. Data were collected in their final year of medical school in 1993/1994 (T1), and in their 10th (T2), 15th (T3) and 20th (T4) postgraduate year (PGY) (2003, 2008 and 2014). Response rates were: 522/631 (83%) at T1, 390/504 (77%) at T2, 330/493 (67%) at T3 and 303/489 (62%) at T4, with all denominator figures being based on mailed out questionnaires. Included in the analyses were responders to all items measuring mastery at the different measurement points. In analyses on low mastery (where mastery was measured at T2, T3 and T4), the number of included responders was 238/522 (46%). In analyses on increased mastery (where mastery was measured as change between T2 and T4), the number of included responders was 256/522 (49%), with all denominator figures being based on responders at baseline/T1. The drop off in the response rates across the ten-year period T2-T4 are due to responders having to respond at all three measurement points to be included.

### Measures

#### Outcome variable

Perceived Mastery of Clinical Work (PMCW) was measured by four items, retained after an exploratory factor analysis of 10 items tapping clinical competence and communication. In substantive terms these four items are very close to the Perceived Mastery of Work four-item scale of the general Nordic Questionnaire for psychological and social factors at work. However out items are more specifically related to clinical work [16]. The four items are: “I have sufficient knowledge and experience to do a good job as a physician”, “I communicate without problems with patients and their next-of-kin”, “I manage to establish collaboration with patients who are poor collaborators to begin with”, and “I experience that I master the professional aspects that my work demands of me”. Responses were on a 7-point Likert scale from 1 = “I agree” to 7 = “I don’t agree”, with scores ranging from 4 to 28. Cronbach’s alphas were 0.88, 0.85 and 0.84 at T2, T3 and T4, respectively.

The cut-off for being low in PMCW was set at the median of each observational point, which was 23 at T2, 24 at T3 and 25 at T4. We defined “increased PMCW” as increasing from below median at T2 to above median at T4.

### Explanatory variables

#### Work-related contextual variables

*Job demands* were measured by an index based on eight items, used and validated in a previous study [19], with questions such as “You have so much influence on your job that you can postpone things that were planned” and “There is sufficient space for you to discuss how to organize your own job”. Since responses on two items were originally on a scale with 4 response categories and those on six items on a scale with 5 response categories, where 1 = seldom/never and 4/5 = daily/often, item raw scores were multiplied with their respective factor score coefficient obtained from the first factor of a principal components analysis at T2. This measure correlates (*r* = 0.37, *P* < 0.001) with Karasek’s demands dimension [19]. (Cronbach’s α = 0.86, 0.87, 0.86).

*Job autonomy* was measured by two items: “To what extent can you control your own work pace?” and “To what extent can you decide or plan the order of your tasks during the day?” Responses were given on a scale with 3 response categories where 1 = to a small extent and 3 = to a great extent. In order to use the same scaling as in former studies [19], we recoded the items in the same way as with the job demands variable. This measure correlates (*r* = 0.53, *p* < 0.001) with Karasek’s control dimension [19]. (Cronbach’s α = 0.81, 0.76, 0.78).

*Job stress* was measured by a modified version of the Cooper Job Stress Questionnaire [20], including four dimensions: *emotional pressure* (Cronbach’s α = 0.85, 0.81, 0.86), *fear of complaints and criticism* (Cronbach’s α = 0.78, 0.74, 0.79), *time pressure* (Cronbach’s α = 0.69, 0.73, 0.72) and *work-home interference* (Cronbach’s α = 0.88, 0.92, 0.91) [21]. This measure has previously been validated [22, 23]. Responses are on a 5-point Likert scale from 1 = Not at all to 5 = Very much.

*Collegial support* was measured by two items: “To what degree do you enjoy working with your colleagues?” and “To what degree are you taken care of by your doctor colleagues?” Responses were on a 7-point Likert scale from 1 = Not at all to 7 = To a very high degree. (Cronbach’s α = 0.84, 0.81, 0.86.) This measure has previously been validated [21, 24].

*Working hours* was measured by one question: “How many hours do you work per week, including all job positions”, with an open response.

*Leading position* was defined as a simple dichotomy: 1 = working as a leader either in a hospital or in general practice, or 0 = all others.

#### Non-work-related contextual variables

*Social support* from family and friends was measured by five items that have been validated elsewhere [25]. The response on one item (“If you have close friends, approximately how often do you talk to them?”) is on a 6-point ordinal scale, which was recoded to a 5-point category scale, where 1 = None or less then every year and 5 = Daily. Responses for the next four items (e.g. “Among those closest to you, is there anyone that is warm, attention giving and interested in what you do?”) were on a 5-point scale, where 1 = Not very/none/unlikely and 5 = Very much/very likely. Answers were reported as a total sum score, with a possible range between 5 and 25. (Cronbach’s α = 0.70, 0.71, 0.74).

*Drinking alcohol to cope with stress* was measured with one question: “When you feel worried tense, or nervous, do you ever drink alcoholic beverages to help you handle things?” Responses were dichotomized as 1 = Seldom, now and then or often, and 0 = Never, as has been accounted for elsewhere [26].

*Taking medicine to cope with stress* was measured with one question: “When you feel worried tense, or nervous, do you ever take medicine to help you handle things?” Responses were dichotomized as 1 = seldom, now and then or often, and 0 = Never.

#### Variables measured during medical school

*Perceived medical recording skills* were measured by six items covering skills in taking a medical history and writing up relevant information from an interview with the patient [27]. Responses were on a 7-point Likert scale from 1 = Never/little to 7 = Always/very much. (Cronbach’s α = 0.77).

*Identification with the role of doctor* was measured by four items on role identification [28]. Responses were on a 7-point Likert scale from 1 = Never/little to 7 = Always/very much. (Cronbach’s α = 0.77).

*Drinking alcohol to cope with stress* was measured in the same way as mentioned above, but assessed during final year in medical school.

*Personality traits* were measured by the Basic Character Inventory, originally constructed by Lazare in 1966 [29] and modified by Torgersen in 1989 [30]. This variable was measured at T1 for half of the sample and one year later for the other half [22]. The 36-item version [23, 28] consists of four personality dimensions. (1) Vulnerability (measuring emotional weakness/dependency/insecurity/“neuroticism”), (2) Intensity (extraversion/affectivity/impulsiveness), (3) Control (obsessiveness/rigidity) and (4) Reality weakness (overwhelming perceptions of the world/thoughts on the borderline between reality and fantasy). Each dimension is based on nine items, with a dichotomous response (agree/do not agree), giving a score range between 0 (low) and 9 (high). (Cronbach’s α: Vulnerability = 0.76, Intensity = 0.77, Control = 0.63, Reality weakness = 0.62 [22]). Since Cronbach’s αs of the two last dimensions are below 0.70 we have excluded those in further analyses.

### Statistical analyses

Kolmogorov-Smirnov and Shapiro-Wilk tests of normality were both significant (e.g. T2: Kolmogorov-Smirnov: 0.17, *P* < .001, Shapiro-Wilk: 0.89, *P* < .001 and T4: Kolmogorov-Smirnov: 0.13, *P* < .001, Shapiro-Wilk: 0.85, *P* < .001), which means that the scores fit the normal curve poorly, and the PMCW scores were slightly skewed. This is however quite common in large samples, and the frequencies of maximum scores were between 5% (at T2) and 13% (at T4). The mean values were very close to the 5% trimmed mean values (e.g. 22.66 vs 22.32 at T2 and 24.84 vs 24.55 at T4), which indicate that the risk of ceiling/floor effects was small. In our analyses, however, the outcome variables are dichotomized, and split at the median, which means that skewness is not a problem. To serve the purpose of this study, we conducted binary logistic regression analyses, starting by studying associations with stable low PMCW. The outcome variable was dichotomized into those being *stable low* in PMCW compared to the others. In these analyses, all contextual explanatory variables, both continuous and dichotomous, were measured by the *mean* value of all measure points (T2, T3 and T4). Hence, answers from dichotomous variables (use of alcohol to cope with stress, use of medicine to cope with stress and being in a leading position) ranged between 0 and 1, where 1 meant a positive answer on all observational points. It was therefore possible to obtain a score for dichotomous explanatory variables between 0 and 1.

When studying associations with increased PMCW over time, the outcome variable was dichotomized as those *increasing* in PMCW compared to the others. In these analyses, all explanatory variables were measured as *change* between T2 and T4.

Explanatory variables such as medical school factors and personality were measured in the final year of medical school. This means that these variables were the only variables measured prior to the outcome in time. Descriptives of all explanatory variables are presented in Table 1, Pearson correlation coefficients between all explanatory variables are presented in Table 2. Problem with multicollinearity are considered low, however the variables Job demands and Job autonomy (*r* = .81, VIF = 4.1–5.0) as well as the Job stress sub-scales: Time pressure and Work-home interference (*r* = .73, VIF = 4.4–5.2) are above the cut off limit of Pearson’s *r* = .70 and VIF = 2.0, indicating some multicollinearity between these variables.

**Table 1:** Mean and range, or percentage, for explanatory variables measured either at T1 or between T2-T4

	Measurement		Mean (SD)	
	Points	N	%	Range
Demographic factors:				
Age	T1	519	28 (2.8)	24–49
Women	T1	522	57%	
Workplace factors:				
Job demands	T2-T4	230	3.8(0.9)	1.7–6.0
Job autonomy	T2-T4	243	2.2(0.6)	1.1–3.3
Job stress: Emotional pressure	T2-T4	185	14.4(3.9)	8–28
Fear of complaints	T2-T4	201	13.3(3.7)	7–24
Time pressure	T2-T4	187	13.0(3.3)	6–25
Work-home interference	T2-T4	231	7.1(2.5)	3–14
Collegial support	T2-T4	236	10.2(1.9)	2–14
Working hours	T2-T4	165	43.3(6.7)	20–62
Leading position^ii^	T2-T4	249	33%	
Non-workplace factors:				
Social support	T2-T4	241	20.4(2.5)	10–25
Alcohol to cope with stress^ii^	T2-T4	247	20%	
Medicine to cope with stress^ii^	T2-T4	247	11%	
Medical school factors:				
Perceived medical recording skills	T1	517	28.6(4.8)	15–42
Identification with role of doctor	T1	516	18.8(4.4)	4–28
Alcohol to cope with stress	T1	520	11%	
Personality:				
Vulnerability	T1^i^	459	3.5(2.3)	0–9
Intensity	T1^i^	459	5.6(2.5)	0–9

SD: Standard deviation of mean, i: half of the sample measured half a year later, ii: % of those reporting “yes” at any of the 3 measuring points

**Table 2:** Pearson correlation coefficient table of explanatory variables

	A	G	JD	JA	EP	FC	TP	WHI	CS	WH	LP	SS	ACS	MCS	PMRS	IRD	ACS	V	I
Age	1	.059	.000	.079	−.013	.020	−.053	−.041	−.065	−.003	.068	−.059	.125	.115	−.003	−.070	.180	−.019	.069
Gender		1	−.059	.069	−.114	−.084	−.043	−.067	−.135	.135	.062	−.245	.173	.055	.082	.103	.075	−.229	−.057
Job demands			1	−.808	.383	.291	.604	.685	−.244	.212	.130	−.244	.239	.194	−.052	−.048	.019	.208	−.129
Job autonomy				1	−.399	−.243	−.511	−.483	.118	−.187	−.108	.124	−.134	−.062	−.024	−.012	.052	−.142	.033
Emotional pressure					1	.617	.633	.419	−.166	−.026	.051	−.126	.095	.158	−.080	−.037	.060	.180	−.093
Fear of complaints						1	.532	.417	−.201	−.032	−.004	−.130	.084	.195	−.200	−.056	.103	.236	−.194
Time pressure							1	.726	−.128	.168	.110	−.172	.105	.094	−.107	.035	−.023	.147	−.064
Work-home interference								1	−.259	.326	.070	−.248	.194	.203	−.102	.033	.016	.219	−.022
Collegial support									1	.191	.103	.564	−.226	−.319	.243	.192	−.112	−.273	.141
Working hours										1	.234	.124	−.034	−.095	.144	.204	−.106	−.008	.011
Leading position											1	−.016	−119	−.133	.154	.108	.067	−.091	.045
Social support												1	−.187	−.185	.252	.211	−.060	−.244	.337
Alcohol to cope with stress													1	.298	−.105	−.013	.295	.110	.032
Medicine to cope with stress														1	−.181	−.112	.238	.211	−.110
Perceived medical recording skills															1	.367	−.032	−.245	.116
Identification with the role of doctor																1	−.048	−.239	.200
Alcohol to cope with stress																	1	.075	.068
Vulnerability																		1	−.188
Intensity																			1
Bold: *p* = < 0.05																			

All unadjusted explanatory variables with *P* < 0.1 were included as candidates for the final adjusted models, in which a stepwise backward elimination procedure was used to arrive at a model containing only significant (*P* < 0.05) explanatory variables. Effects were assessed by odds ratio and the total explained variance of PMCW in each final model was indicated by both Cox & Snell R^2^ and Nagelkerke R^2^. Interaction analyses were used to assess whether any of the explanatory variables showed significantly different effects between the two genders.

### Missing data

Included in the analyses were responders to all four items measuring the PMCW variable, as well as responders to all items of all explanatory variables, except for the four personality dimensions. To reduce sample attrition because of lacking responses to some of the personality items, scores for items in a given personality dimension were imputed with mean scores when responses were missing for 4 or fewer of the 9 items. An attrition bias test was performed by comparing responders at T1 with those who actually answered all items of the PMCW variable at T2, T3 and T4 respectively. This showed a significant difference in age between responders and non-responders at both T2 and T3, where non-responders were 0.8 years older than responders at T2 (*r* = 0.13, *P* = 0.004) and 0.5 years older at T3 (*r* = 0.09, *P* = 0.04). In addition, there was a significant difference in responses to the identification with the role of doctor scale between responders and non-responders at T3, where non-responders answered 0.8 scores lower (*r* = − 0.09, *P* = 0.04) than responders on the role identification scale (range 4–28). There were no significant differences between responders and non-responders for the PMCW variable at T4. Thus, there were few indications of skewed attrition for the sample as whole.

## Results

### Characteristics of stable low PMCW between 10th and 20th PGY

Stable low perceived mastery was reported by 31% (*n* = 73) of the doctors. Unadjusted significant associations with stable low PMCW are shown in the first column in Table 2. Adjusted statistically significant associations were the personality trait of *vulnerability* (OR: 1.30, *P* < .000, CI: 1.12 to 1.50), drinking *alcohol to cope* with stress during medical school (OR: 2.66, *P* = .04, CI: 1.03 to 6.85) and *social support* from family and friends (OR: 0.78, *P* = .002, CI: 0.66 to 0.91). The final model explained between 18% (Cox & Snell R^2^) and 26% (Nagelkerke R^2^) of the variance in PMCW (Table 3).

Interaction analyses revealed no interaction between any of the significant explanatory variables, but showed significant interaction between gender and working hours (*P* = 0.03) and between gender and use of medicine to cope with stress (*P* = 0.049).

### Characteristics of increased PMCW between 10th and 20th PGY

**Fig. 1:**
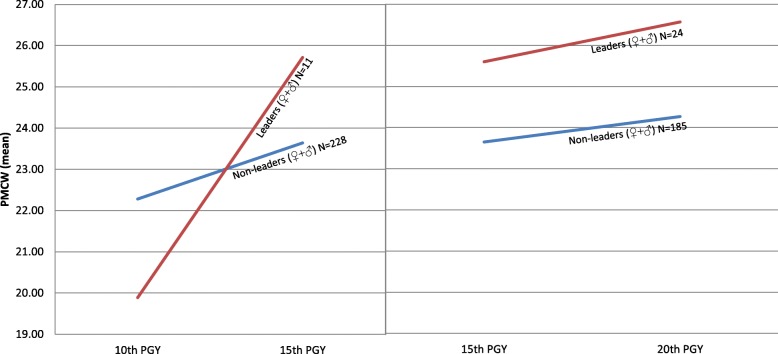
Mean value of PMCW for leaders vs non-leaders between 10th and 15th PGY^*^ and between 15th and 20th PGY^*^. ^*^Controlled for all other significant explanatory variables

Increased perceived mastery was reported by 15% (*n* = 39) of the doctors. The only unadjusted explanatory variable of increased PMCW was perceived *job demands*. In the adjusted model, however, due to possible statistical suppression, both taking up a *leading position* (OR: 3.04, *P* = .01, CI: 1.31 to 7.07) and perceived *job demands* (OR: 0.66, *P* = .02, CI: 0.45 to 0.98) were significant adjusted explanatory variables. The final model explained between 7% (Cox & Snell R^2^) and 12% (Nagelkerke R^2^) of the variance in PMCW (Table 3). Mean values of PMCW at the three measurement points for leaders and non-leaders are presented in Figure. 1.

**Table 3:** Characteristics of low and increased Perceived Mastery of Clinical Work between 10th and 20th PGY

	Characteristics (measured as mean) of low PMCW^a^				Characteristics (measured as change) of increased PMCW^b^			
	Unadjusted		Adjusted		Unadjusted		Adjusted	
	OR	CI 95%	OR	CI 95%	OR	CI 95%	OR	CI 95%
Demographic factors^c^:								
Age	1.00	0.88 to 1.13	e		1.00	0.87 to 1.15		
Gender	1.49	0.86 to 2.60	e		0.98	0.49 to 1.96		
Workplace factors^d^:								
Job demands	1.12	0.80 to 1.57			0.64^*^	0.45 to 0.92	0.66^*^	0.45 to 0.98
Job autonomy	0.85	0.53 to 1.36			1.40	0.87 to 2.26		
Job stress: Emotional pressure	1.03	0.95 to 1.12			1.05	0.97 to 1.15		
Fear of complaints	1.04	0.96 to 1.13			1.03	0.95 to 1.11		
Time pressure	1.00	0.90 to 1.09			0.94	0.86 to 1.04		
Work-home interference	1.03	0.92 to 1.16			0.92	0.83 to 1.02		
Collegial support	0.69^***^	0.58 to 0.82	e		1.05	0.91 to 1.22		
Working hours	0.96	0.91 to 1.01			0.97	0.94 to 1.00	e	
Leading position	0.49	0.16 to 1.54			2.00	0.98 to 4.09	3.04^*^	1.31 to 7.07
Non-workplace factors^d^:								
Social support	0.77^***^	0.68 to 0.88	0.78^**^	0.66 to 0.91	1.00	0.88 to 1.14		
Alcohol to cope with stress	1.67	0.58 to 4.77			1.75	0.62 to 4.92		
Medicine to cope with stress	2.95	0.65 to 13.51			0.56	0.16 to 1.92		
Medical school factors^c^:								
Perceived medical recording skills	0.90^**^	0.84 to 0.96	e		1.01	0.94 to 1.10		
Identification with the role of doctor	0.87^***^	0.81 to 0.93	e		1.01	0.93 to 1.09		
Alcohol to cope with stress	3.36^**^	1.44 to 7.85	2.66^*^	1.03 to 6.85	1.26	0.45 to 3.56		
Personality^c^:								
Vulnerability	1.32^***^	1.16 to 1.50	1.30^***^	1.12 to 1.50	0.94	0.81 to 1.10		
Intensity	0.88^*^	0.78 to 0.98	e		1.03	0.90 to 1.19		
Control	0.99	0.86 to 1.14			1.06	0.90 to 1.25		
Reality weakness	1.36^**^	1.11 to 1.67			1.00	0.79 to 1.27		

OR: Odds Ratio, *: *p* = < 0.05, **: *p* = < 0.01, ***: *p* = < 0.001, a: Cox&Snell R^2^: .18, Nagelkerke R^2^: .26, b: Cox&Snell R^2^: .07, Nagelkerke R^2^: .12, c: measured at T1, d: measured by mean value of all measuring points in each time period in analysis on low PMCW and measured as change between T2 and T4 in analysis on increased PMCW, e: Variables included in the adjusted model due to *p* = < 0.1, but removed through stepwise backward elimination

Interaction analyses revealed no interactions with any of the significant explanatory variables, but showed significant interaction with gender and change in use of alcohol to cope (*P* = 0.03).

## Discussion

The aim of this study was to investigate characteristics of doctors with stable low levels and with increased levels of perceived mastery over a period of ten years of medical practice. Stable low perceived mastery over ten years of practice was reported by 31% of the doctors. Reporting stable low perceived mastery was associated with the personality trait of vulnerability, drinking alcohol to cope with stress during medical school and low perceived social support.

Increased perceived mastery was reported by 15% and was associated with taking up a leading position and with a decline in perceived job demands.

### Characteristics of doctors with low perceived mastery over ten years of practice

The vulnerability scale includes difficulties in handling negative criticism and a belief that others do things better than oneself [30]. With such a self-image, challenging oneself in demanding situations – with the risk of experiencing failure or being criticized – may be avoided. If a vulnerable personality leads to the tendency not to put oneself in new demanding situations, this could explain the association with decreased sense of mastery [31, 32].

Similarly, using alcohol to cope with stress during medical school could be a sign of an unhealthy avoidant behavior pattern [33]. Alcohol abuse has also been linked to both burnout and lower career satisfaction among doctors [34]. A previous study by our research group showed that use of alcohol to cope with stress during medical school was significantly associated with lower perceived mastery early in the career but not later [16]. In contrast to these findings, the present study indicates that this risky avoidant behavior could be associated with low perceived mastery for as long as 20 years after graduation. Explanations as to why this unhealthy coping strategy is used by those individuals should therefore be further explored.

If put in a demanding situation, we tend to feel less stressed or anxious when surrounded by people that are close to us, and that we trust [35]. One of the three components of Antonovsky’s sense of coherence, viz. manageability, includes a sense of being able to handle difficult situations on one’s own, but also a belief that one can rely on close ones in difficult situations [17]. Low perceived collegial support was significantly associated with low mastery in the unadjusted model, but not in the adjusted, while perceived social support was significant in both models. We have no evidence to link collegial and social support, but low scores on both might indicate a kind of passive social behavior, and be a sign of not belonging to a social group and thus lacking coping resources in difficult situations.

On the basis of our findings, we would propose that the factors characterizing those reporting stable low mastery over time could be interpreted as long-term correlates of passive or avoidant behavior. We believe that low mastery could be a negative inner reinforcement that increases an avoidant behavior, as explained in e.g. theories of cognitive behavioral therapy [33, 36]. Vulnerability as well as use of alcohol to cope with stress during medical school and low social support could in this respect be both the cause and effect of an avoidant coping behavior.

### Characteristics of increased perceived mastery over ten years of practice

Challenging work (followed by a sense of accomplishment) has shown an association with higher sense of mastery of work [37]. In our study, we see that doctors in leading positions start off on a rather low level of mastery in their 10th PGY, but markedly increase their sense of mastery already by the 15th PGY. We see the same tendency for both genders. Thus, being in a leading position early in the career does not seem to increase one’s sense of mastery in itself. It could perhaps rather be a sign of being placed in a demanding situation, with a concomitant sense of lacking mastery of work at the time. Remaining in a leading position, however, and gaining the experience of being able to handle the situation could result in a higher sense of mastery over time. Yet it could also be the other way round, namely that increased sense of mastery makes a person want to stay in a leading position.

Exposing oneself to demanding situations most probably changes one’s perceptions of such situations, while trying to change one’s perception of a situation is more difficult if one does not work with behavior change simultaneously [33]. Adding cognitive aspects to behavioral change shows little effect [38, 39]. Taking up leading position could be a behavior in line with the tendency of daring to put oneself in a possibly uncomfortable demanding situation, and doing so to develop in one’s profession despite the risk of being criticized. This is in contrast to the underlying component of the personality trait of vulnerability: fear of criticism. The findings do indicate a link between leadership career trajectories and increased perceived mastery of work. It is, however, important to note that taking up a leading position can also be a simple choice of career, and does not necessarily mean that the individual has a general proactive behavior.

A decline in sense of job demands is indeed in line with former studies on associations with perceived mastery [10, 11], and also with our hypothesis; it could be a natural result of having the feeling of being able to handle demanding work-related situations – the result of having a sense of mastery of work. Again, it is possible that the association goes the opposite way: that a decline in perceived job demands leads to increased sense of mastery of work.

In contrast to previous studies, we did not find any associations between social or collegial support and increased mastery [1, 11]. The lack of associations could be due to a Type II error, since only *N* = 39 doctors increased their sense of mastery during the ten years of practice. Nevertheless, this should be studied further.

### Implications for the individual doctor and for the workplace

The low number (15%) reporting increased sense of mastery from low to high during the ten year period of measurement, does not say anything about those reporting stable high sense of mastery during the whole time period or those increasing their mastery from high to a little higher, or indeed from very low to moderate (but still below the median). However, this is a group that may give important information about how one could help individuals to increase an initially low sense of mastery of their clinical work already in medical school. Exposing oneself to more stress or challenges or exposing oneself to the risk of being criticized could be important in order to develop higher mastery or self-efficacy for the individual doctor. These mechanisms should be taught and learned by students already in medical school. Factors associated with low perceived mastery could be construed as possible passive or avoidant behavior. This implies creating a working environment that promotes proactive social behavior and healthy coping strategies among the employees. The so-called Balint groups, introduced in the 1960s [40], constituted a forum where doctors could develop their abilities in seeking advice from colleagues, and experience social support from others. Re-introducing these groups, or something similar, could benefit the individual doctor’s personal development and well-being [41], which in the long term could benefit work performance [8, 9].

### Strengths and limitations

The main strengths of this study include the 20-year longitudinal design with a nationwide sample, the relatively high response rates, and the small differences between responders and non-responders. The use of three observation periods increases the reliability of the explanatory and contextual variables. The study does have several limitations, including data being based solely on self-report and the cut-off on our outcome variable lacking validation elsewhere. We also lacked the possibility to check for any attrition bias on non-responders at the very first measuring point. Personality was measured in the final year of medical school (and for half of the sample one year later), and is not necessarily stable over 20 years. The variable “taking medicine to cope with stress” can be argued to be unspecific as the type of medicine could be relevant. With this study design, we can only hypothesize about the cause and effect of the associations found between perceived mastery and our explanatory variables. Since we have no data on perceived mastery of clinical work measured at the final year of medical school, we can never know if the individuals reporting high vulnerability or the use of alcohol to cope with stress during medical school would also report low perceived mastery already at that point. We can therefore not be sure that these variables are “causes” of low perceived mastery later in the career. We can conclude with these factors being *associated* with each other, but future research needs to study the causality between these factors. This is also the case with contemporary measured significant explanatory variables measured at the same time as perceived mastery. We found an *association*, but we cannot conclude that our explanatory variables do in fact cause low or increased perceived mastery, or vice versa.. The question regarding being in or obtaining a leading position is somewhat narrow given that increased perceived mastery is a major focus of the study yet increased perceived mastery was only reported by 15% of the doctors. Future research should also investigate possible associations with specialties.

## Conclusion

Having a vulnerable personality, a history of having used alcohol to cope with stress during medical school and lack of contemporary social support are all associated with stable low mastery over time. Conversely, taking up a leading position and having the perception that job demands are decreasing over time are associated with an increased sense of clinical mastery. These findings indicate that perceived mastery of clinical work may not be a trait, but modifiable over time.

## Abbreviations

NORDOC: The Longitudinal Study of Norwegian Medical Students and Doctors
PGY: Postgraduate year
PMCW: Perceived Mastery of Clinical Work

## References

[1] Bennetter KE (2016). Clench–Aas J, Raanaas RK. Sense of mastery as mediator buffering psychological distress among people with diabetes. J Diabetes Complicat.

[2] Nygren B, Aléx L, Jonsén E, Gustafson Y, Norberg A, Lundman B (2005). Resilience, sense of coherence, purpose in life and self-transcendence in relation to perceived physical and mental health among the oldest old. Aging Ment Health.

[3] Taylor C, Graham J, Potts HWW, Richards MA, Ramirez AJ (2005). Changes in mental health of UK hospital consultants since the mid-1990s. Lancet.

[4] Mosley TH, Perrin SG, Neral SM, Dubbert PM, Grothues CA, Pinto BM (1994). Stress, coping and well-being among third-year medical students. Acad Med.

[5] Prins JT, Hoekstra-Weebers JEHM, van de Wiel HBM, Gazendam-Donofrio SM, Sprangers F, Jaspers FCA (2007). Burnout among Dutch medical residents. Int J Behav Med.

[6] Grassi L, Magnani K (2000). Psychiatric morbidity and burnout in the medical profession: an Italian study of general practitioners and hospital physicians. Psychother Psychosom.

[7] Shanafelt TD, Hasan O, Dyrbye LN, Sinsky C, Satele D, Sloan J (2015). Changes in burnout and satisfaction with work-life balance in physicians and the general US working population between 2011 and 2014. Mayo Clin Proc.

[8] Shanafelt TD, Bradley KA, Wipf JE, Back AL (2002). Burnout and self-reported patient care in an internal medicine residency program. Ann Intern Med.

[9] Fahrenkopf AM, Sectish TC, Barger LK, Sharek PJ, Lewin D, Chiang VW (2008). Rates of medication errors among depressed and burnt out residents: prospective cohort study. BMJ.

[10] Karasek R, Theorell T (1990). Healthy work: stress, productivity and the reconstruction of working life.

[11] Ljoså CH, Tyssen R, Lau B (2013). Perceived mastery of work among shift workers in the Norwegian offshore petroleum industry. Ind Health.

[12] Siltaloppi M, Kinnunen U, Feldt T (2009). Recovery experiences as moderators between psychosocial work characteristics and occupational well-being. Work & Stress.

[13] Pearlin LI, Nguyen KB, Schieman S, Milkie MA (2007). The life-course of mastery among older people. Soc Behav.

[14] Fritz C, Sonnentag S (2006). Recovery, well-being and performance-related outcomes: the role of work load and vacation exeriences. J Appl Psychol.

[15] Binnewies C, Sonnentag S, Mojza EJ (2009). Feeling recovered and thinking about the good sides of one’s work. J Occup Health Psychol.

[16] Belfrage A, Støen Grotmol K, Lien L, Moum T, Wiese RV, Tyssen R. Medical school predictors of later perceived mastery of clinical work among Norwegian doctors: a cohort study with 10 and 20 year follow-up. BMJ Open. 2017; In press. 10.1136/bmjopen-2016-014462 PMC5623388 28947437

[17] Antonovsky A (1987). Unraveling the mystery of health: how people manage stress and stay well.

[18] Støen Grotmol K, Gude T, Moum T, Vaglum P, Tyssen R (2013). Risk factors at medical school for later severe depression: a 15-year longitudinal, nationwide study (NORDOC). J Affect Disord.

[19] Mahmood JI, Støen Grotmol K, Tesli M, Vaglum P, Tyssen R (2017). Contextual factors and mental distress as possible predictors of hazardous drinking in Norwegian medical doctors: a 15-year longitudinal, nationwide study. Eur Addict Res.

[20] Cooper CL, Rout U, Faragher B (1989). Mental health, job satisfaction, and job stress among general practitioners. BMJ.

[21] Rovik JO, Tyssen R, Hem E, Gude T, Ekeberg O, Moum T (2007). Job stress in young physicians with an emphasis on the work-home interface: a nine-year, nationwide and longitudinal study of its course and predictors. Ind Health.

[22] Tyssen R, Vaglum P, Grønvold NT, Ekeberg Ø (2000). The impact of job stress and working conditions on mental health problems among junior house officers. A nationwide Norwegian prospective cohort study. Med Educ.

[23] Tyssen R, Vaglum P, Grønvold NT, Ekeberg Ø (2005). The relative importance of individual and organizational factors for the prevention of job stress during internship: a nationwide and prospective study. Med Teach.

[24] Hertzberg TK, Isaksson Rø K, Vaglum P, Moum T, Røvik JO, Gude T (2016). Work-home interface stress: an important predictor of emotional exhaustion 15 years into a medical career. Ind Health.

[25] Tyssen R, Hem E, Gude T, Grønvold N, Ekeberg Ø, Vaglum P (2009). Lower life satisfaction in physicians compared with a general population sample. Soc Psychiatry Psychiatr Epidemiol.

[26] Mahmood JI, Grotmol KS, Tesli M, Vaglum P, Tyssen R (2016). Risk factors measured during medical school for later hazardous drinking: a 10-year, longitudinal, nationwide study (NORDOC). Alcohol Alcohol.

[27] Tyssen R, Vaglum P, Grønvold NT, Ekeberg Ø (2001). Factors in medical school that predict postgraduate mental health problems in need of treatment. A nationwide and longitudinal study Med Educ.

[28] Gude T, Vaglum P, Tyssen R, Ekeberg Ø, Hem E, Røvik JO (2005). Identification with the role of doctor at the end of medical school: a nationwide longitudinal study. Med Educ.

[29] Lazare A, Klerman GL, Armor DJ (1966). Oral, obsessive, and hysterical personality patterns: an investigation of psychoanalytic concepts by means of factor analysis. Arch Gen Psychiatry.

[30] Torgersen S, Alnæs R (1989). Localizing DSM-III personality disorders in a three-dimensional structural space. J Personal Disord.

[31] Bandura A (1977). Self-efficacy: toward a unifying theory of behavioral change. Psychol Rev.

[32] Knardahl S. Mastery of work. Review of psychological and social factors at work and suggestions for the general Nordic questionnaire (QPS Nordic). Copenhagen: Nordic Council of Ministers. 1997:63–6.

[33] Ramnerö J, Törneke N (2006). Beteendets ABC : en introduktion till behavioristisk psykoterapi [the ABC of behavior: an introduction to behavioral psychotherapy]. Lund: Studentlitteratur.

[34] Oreskovich MR, Shanafelt T, Dyrbye LN (2015). The prevalence of substance use disorders in American physicians. Am J Addict.

[35] Kikusui T, Winslow JT, Mori Y (2006). Social buffering: relief from stress and anxiety. Philos Trans R Soc Lond Ser B Biol Sci.

[36] Andersson LE, Klintrot M (2013). OBM - Ledarskapets psykologi: För chefer, ledare, projektledare och andra som arbetar med människor [OBM – Leadership psychology: For managers, leaders, project managers and others working with people].

[37] Bradley GL (2010). Work-induced changes in feelings of mastery. Aust J Psychol.

[38] Dimidjian S, Hollon SD, Dobson KS, Schmaling KB, Kohlenberg RJ, Addis ME (2006). Randomized trial of behavioral activation, cognitive therapy, and antidepressant medication in the acute treatment of adults with major depression. J Consult Psychol.

[39] Öst L-G, Thulin U, Ramnerö J (2004). Cognitive behavior therapy vs exposure in vivo in the treatment of panic disorder with agrophobia. Behav Res Ther.

[40] Balint M (1957). The doctor, his patient and the illness.

[41] Kjeldmand D, Holmström I (2008). Balint groups as a means to increase job satisfaction and prevent burnout among general practitioners. Ann Fam Med.

